# A Case

**Published:** 1862-12

**Authors:** L. B. Brown

**Affiliations:** Iroquois, Ills.


### A CASE.

By L. B. BROWN, M. D., Iroquois, Ills.

Messrs. Editors of the Chicago Med. Journal:

I was called in July, 1861, to see a lad about thirteen years old, with fractured *Clavicle.* The accident occurred some two or three days previous to my visit, and had been treated by a "physician" living in town. The case, so far as the fracture alone was concerned, presented nothing unusual, but the manner in which it was dressed, and, particularly the operation to be performed by the attendant, (who was momentarily expected,) was astonishing to me as related by the mother of the patient.

The mountebank, after making use of all means known to him (except heroic) whereby fractures are to be adjusted, declared that it was necessary to make an incision through the skin, and introduce a hook in order to raise the rebellious portion of the clavicle. It was in consequence of this declaration of the quack that I was called.

After disengaging the patient from an almost inextricable application of compresses and bandages which were sewed at every accessible point, and seemed applied for the double purpose of *securing the patient* and keeping him warm, I applied the common figure of 8 bandage, with pad in the axilla, and sling. A cure was completed in five or six weeks without difficulty.

Question—Is it advisable to report such cases?

